# Well-Being without Employment? Promoting the Employability of Refugees

**DOI:** 10.3390/ijerph17217775

**Published:** 2020-10-23

**Authors:** Lucía I. Llinares-Insa, Manuel Roldán-Pardo, Pilar González-Navarro, María Desamparados Benedito-Monleón

**Affiliations:** 1 Facultat de Psicologia, Universitat de València, 46010 València, Spain; lucia.llinares@uv.es (L.I.L.-I.); rolparma@uv.es (M.R.-P.); abenedit@uv.es (M.D.B.-M.); 2 Research Institute of Human Resources Psychology, Organizational Development and Quality of Working Life (IDOCAL), Universitat de València, 46010 València, Spain

**Keywords:** employability, refugee, intervention, labor market, social inclusion

## Abstract

There are more than 25 million refugees in the world. Many of them try to reach the Mediterranean in order to enter Europe. Spain is one of the countries that receive refugees and have to integrate them. Many refugees have experienced persecution in their countries, as well as forced migration, rape, diseases, etc. Their integration requires support and coordination from the government, health services, and social agents. The first step in achieving this integration is getting a job, which is currently an important issue. Thus, we aim to analyze the employability of a specific group of refugees in Spain and then develop and implement an intervention program to improve their employability. Our framework is based on the Bioecological Model of Employability. The results obtained show that the program is effective in improving employability, and they highlight the importance of labor inclusion for refugees’ well-being. Moreover, the findings reveal the need to create labor market policies and further evaluations, diagnostics, and intervention programs that improve employability and other types of personal-community growth. It is necessary to focus on refugees’ needs and develop appropriate services.

## 1. Introduction

Active inclusion is a concept the European Commission [1] emphasizes for the labor market inclusion of unemployed or/and vulnerable people. In EU member states, there is support for these labor market policies [2].

Understanding the employability of vulnerable people is important because it can suggest ways for social agents to carry out interventions. Moreover, employability is a tool for analyzing and modifying the labor, socioeconomic, and political context [3]. The European Union (EU) presents employability as the path to full employment and active citizen promotion [4], and as a fundamental strategy to reduce current unemployment rates [5] and poverty [6,7]. In fact, employability is a mechanism to reduce job insecurity and improve job welfare [8]. Therefore, employability is an important issue in the current scenario and deserves more attention [9,10].

Currently, getting a job can be defined as an evolutionary process because it allows social integration, provides economic stability, and promotes health [11]. However, labor market conditions and the current economic crisis in the Western world make this job-seeking and job-obtaining process difficult. The effects of this deep crisis in Spain can be seen, for example, in the destruction of jobs and the increasing precariousness of employment. The conditions of the labor market and the current situation of the global economic crisis make it difficult for refugees in particular to find and obtain work. Thus, economic, social, and territorial inequalities are accentuated [12]. Instability, discrimination, alterations in health, and unworthy living conditions are characteristics of the current society in which we live. Therefore, job insecurity translates into social and economic discrimination, health diseases, and unsuitable living conditions, etc. (see [13]). For refugees, getting a job is necessary for their social integration, and employability is their best resource.

The scientific literature shows many definitions of employability, and it is difficult to find a consensual one. For some authors, employability is the capacity for self-movement within the labor market, in order to apply one’s potential to lasting jobs [14]. For other authors, employability is the personal capacity or opportunity to find a job, either in the first job-seeking situation or when trying to find another alternative job [8,15,16]. Moreover, Van der Heijde and Van der Heijden [17] refer to employability as a systematic ability to obtain or create work through the optimization of personal skills.

In all these definitions, the construct refers to the person and society. However, we need a framework with a holistic view that integrates the individual’s responsibility, personal circumstances, and contextual factors of employability. The Bioecological Model of Employability [18] provides this framework, based on the bioecological theory [19]. In this model, employability is defined as a transversal meta-competence related to employment [20] that includes thirteen indicators: perseverance, academic qualifications, professional qualifications, learning to learn, time management, task management, initiative, social skills, autonomy, the will and willingness to work, specific professional skills, personal care, and work experience [3].

In the scientific literature, studies have analyzed employability in university students (e.g., [21]), older workers (e.g., [22]), and according to gender (e.g., [23]), but migrant workers (e.g., [24]) or refugees are not usually studied. However, according to Álvarez, Favieres, Muñiz, Senante, Valiente, and Amorós [25], there are 70 million refugees in the world. The European Union member states do not unitarily confront the challenges of forced migration [26], and the Mediterranean is the most perilous migration route. In 2019, 118,264 people requested international protection in Spain, which represents an increase of 118.7% compared to 2018. Moreover, Spain is one of the European Union countries in which poverty has grown the most, and job insecurity rates have reached 40% [25]. 

For the UN Refugee Agency (UNHCR), refugees are people fleeing their countries due to armed conflicts or persecution. Their situation is both dangerous and intolerable, given that they escape from their country to search for security, assistance, and recognition as refugees [27]. This recognition is necessary because it is too dangerous for these people to return to their country of origin [28]. 

Different authors have pointed out that participation in the labor market is the most important factor in refugees’ social integration and health and well-being. However, there is a ‘refugee gap’ [29] that is reduced over time and through labor inclusion (active job) in the receiving countries [30]. Blustein [31] highlights that being part of the working population involves social recognition and provides resources for a dignified life, sustainable development, and health and well-being. This situation makes it possible “to live productively, happily and healthily” [32] (p. 308). However, in most EU countries, refugees are excluded from participation in the labor market [26]. Correa–Velez, Barnett, and Gifford [33] state that entering the labor market is difficult for refugees and that some of them experience high social barriers (legal regulations, few bridging institutions between them and their labor integration, racism, a lack of experience with the labor market of the host country, and missing employability skills for this country). Desiderio [34] used the expression ‘missing link’. This situation has detrimental effects on health, personal security, self-esteem, and overall well-being [35].

The European Quality Assurance Reference Framework for Vocational Educational and Training (VET) has been adopted by EU countries [36]. It aims to improve the employability of vulnerable groups such as adult refugees [37]. In Spain, some nonprofit organizations currently defend the need to increase employability skills (e.g., [38]), and the European Commission highlights the importance of refugees’ employability for their labor market integration [39]. In this line, the labor integration of refugees is urgent. They make up a smaller population than immigrants but have a greater vulnerability. Specifically, according to the European Training Foundation (ETF) [40], since 2008, Southern Mediterranean countries such as Spain have produced uneven growth, low-quality jobs, and high unemployment among vulnerable people. 

The data provided by Gayo and Quintana [41] showed that job destruction and a high concentration in a few labor sectors have led to an increase in the number of unemployed individuals in this collective, decreasing their employment contracts and work authorizations and lowering salaries. Therefore, the poverty risk rate has increased exponentially in this social group. All these aspects have contributed to aggravating what some experts have called ‘the refugee crisis’ (see [42]).

Thus, due to the relevance of changing this situation, our aim is to analyze refugees’ employability in Spain and to implement some intervention strategies to improve it. 

Well-being is an indicator of positive psychological development, and it is positively linked to employability (e.g., [43]), career success [44], and inclusion in the active labor market of the receiving countries. It is related to individual characteristics in the acquisition of employability skills (e.g., flexibility) [45] and contextual dimensions [46]. This study, in line with Bronfenbrenner’s Bioecological Theory, seeks a comprehensive and manageable approach to the development of refugees’ employability. For this purpose, the theoretical framework used is the Bioecological Model of Employability (BME) by Llinares, Zacarés, and Córdoba [18]. Thus, we assess refugees’ employability in one of the Spanish CARs (Refugee Reception Centers) using the EAS (Employability Appraisal Scale by Llinares, González, Zacarés, and Córdoba) [3]. 

The results will allow us to design, implement, and evaluate interventions to improve refugees’ employability. Hence, refugees will be able to improve their employability meta-competence in order to effectively manage their own labor careers. 

Some intervention programs have been developed to teach employability-communication skills to adult migrants (e.g., [47,48]), and there are projects to increase migrants’ employability (e.g., project SAMIN in SOLIDAR [49]). However, few studies and interventions have focused on refugees’ employability, even though they make up a high-risk group with serious job-seeking difficulties. They are forced to accept precarious job conditions for which they might be overqualified [50]. Moreover, most of these studies have focused on refugees hosted by classic receiving countries, such as the United States, Canada, and Australia [51,52,53]. Their findings showed that refugees have an economically disadvantageous position compared to other social groups. 

Some recent studies have been interested in health and integration policy aspects. Väyrynen [54] states that health is an important factor in explaining economic disadvantage because refugees have experienced a post-traumatic stress situation. In addition, authors also state that the predisposition of the integration policies can make the difference between social inclusion and marginalization [53].

De Vroome and Van Tubergen [55] analyzed four factors in western European countries, and they found, regarding human capital, that knowledge of the host country language and work experience played a very important role. Moreover, education in the host country plays a more relevant role in labor and economic inclusion than the education received in the country of origin. Finally, they found an international transferability phenomenon [56,57]. In other words, education stimulation and development opportunities involved the reactivation of skills and abilities that already existed in the person but were not manifested in the receiving country. Regarding social capital, contact with associations or institutions and the native population plays a fundamental role in economic and labor integration, as well as contact—oriented towards employability—with the ethnic group and place of residence [58]. In the health area, general health problems and depression negatively influence employability. Böckerman and Ilmakunnas [59] have highlighted that unemployment and health problems constitute a vicious circle that feeds itself continually because unemployment is associated with health problems and health problems lead to unemployment. Unemployed refugees have economic difficulties and a lack of access to basic resources for their social integration. Campbell, Mann, Moffatt, Dave, and Pearce [60] reported that this situation was related to poor emotional well-being and health. Therefore, a higher unemployment and poor health usually have interrelated effects in refugees [61]. Finally, based on the receiving country’s policies, there is an inversely proportional relationship between staying in institutional centers and labor and economic integration. However, this relationship is mediated by post-migratory human capital; that is, a greater human capital is related to a less negative impact on labor and economic integration [55].

A comprehensive approach to the refugees’ problem is needed from an employability perspective as a possible tool to improve their precarious situation and social exclusion risk. Therefore, we aim to develop and implement an effective and efficacious psychosocial intervention program to improve the employability skills of refugees in Spain. Based on an early-stage diagnostic assessment to identify refugees’ employability skills, the main hypothesis is that the intervention would significantly improve refugees’ employability and impact their labor and economic integration [55]. 

## 2. Study 1: Population Needs Assessment

### 2.1. Method

#### 2.1.1. Participants

The sample was composed of immigrants from a Spanish refugee reception center. A Refugee Reception Center [CAR] is a tool that serves the basic needs and social integration of the applicants or beneficiaries of international protection with a stateless or temporary protection status. Therefore, they are public establishments that depend on the Ministerio de Empleo y Seguridad Social. These centers provide accommodation, maintenance, urgent and primary psychosocial assistance, and other social services, and their main purpose is to facilitate coexistence and integration in the Spanish community. Under L.O. 4/2000 [62], few centers perform this service in our country, and so it is important to intervene to increase employment skills.

However, not all asylum seekers can benefit from these services. Those who meet any of the following conditions can be beneficiaries: (a) have requested to be or are a beneficiary of international protection in Spain; (b) have the document proving their status as an applicant or beneficiary of international protection, or have a stateless or temporary protection status; or (c) have requested international protection in one of the European Union states ruled by Regulation 343/2003 of the European Council of February 18 and not been admitted to process this request. In addition, CAR Mislata has some other conditions: (a) not having work or economic resources to take care of their own needs or those of their family; (b) not having any contagious disease or type of disability (physical or psychological) that can disrupt the coexistence in the center; and (c) accepting the center’s regulations.

The Refugee Reception Center that was analyzed contained 12 people (*n* = 12). The average age was 25.58 years (SD = 4.89), ranging between 19 and 35 years, and 25% were women (*n* = 3, Mage = 26, SD = 7.81). These women lacked education because they did not finish primary school. Two of them were single, and the other was married. Men represented 75% (*n* = 9, Mage = 25.44, SD = 4.19). In this case, there was more variation in the education levels: 22% had no studies, 11% had primary studies, 44% had secondary studies, and 23% had university studies. Regarding their marital status, 66% were single, 22% were married, and the remaining 12% were divorced.

Additionally, we interviewed the institution’s social agents (*n* = 3), three social intervention professionals who worked in the center. These workers were the employment technician, the social worker, and the psychologist.

#### 2.1.2. Instruments

A questionnaire battery was used that included sociodemographic, labor mobility (understood as the ability to move to other cities and work in them), and work experience variables.

Employability was analyzed with the Spanish version of the Employability Appraisal Scale (EAS) [3]. It is a validated questionnaire that measures individual and contextual aspects of employability. The EAS contains 35 Likert-type items, where 1 corresponds to ‘never/nothing’ and 5 corresponds to ‘always’. The scale assesses thirteen significant employability indicators: perseverance, academic qualifications, professional qualifications, learning to learn, time management, task management, initiative, social skills, autonomy, the will and willingness to work, specific professional skills, personal care, and work experience. It is composed of five subscales: Employment protective behavior, Employment risk, Job-seeking behavior, Self-control, and Self-learning. The reliability indexes, Cronbach alphas, were adequate, except for the Employment risk index (α Employment protective behavior = 0.71, α Employment risk = 0.29, α Job-seeking behavior = 0.61, α Self-control = 0.51, and α Self-learning = 0.8). 

Factor 1, employment protective behavior, was related to the work behaviors of persistence, responsibility, planning and organization, maintenance, and progress in the process of labor insertion; it was also related to expectations of self-efficacy in the achievement of goals and confidence in one’s criteria. Factor 2, employment risk, was related to different individual aspects (planning and organizational skills) and requirements for work (training and experience). Factor 3, job-seeking behavior, was related to the active behavior and skills to find a job. Factor 4, self-control, was related to emotional control (anger, frustration, rage). Factor 5, self-learning, was related to learning to learn at work and improving labor skills.

Moreover, we asked the CAR professionals for a semi-structured interview. The steps proposed by Vallés [63] were followed in its design. The place, time, and means of registration, written through notes, were agreed upon.

#### 2.1.3. Procedure

We went to the CAR on a Thursday afternoon because a conference was taking place and all the users were there. In this conference, we were given space to present the program, and afterwards, those who were interested stayed to fill out the questionnaires. 

The measurement instruments were administered in one of the multipurpose rooms in the center. In all cases, the questionnaires were in a paper and pencil format, and their implementation was individual, without including any personal details that could identify them. Anonymity was guaranteed. However, refugees do not fluently read Spanish, and so we had to present the items in a structured interview.

The interviews with the professionals were carried out in their respective offices at a time that they proposed. Participation was completely confidential and voluntary. The interviews were conducted in a relaxed manner, covering the planned topics as well as other topics of interest suggested by the professional. The interviews were not videotaped. They lasted about 15 to 20 min.

#### 2.1.4. Data Analysis

Quantitative and qualitative analyses were performed. The former was implemented through the statistical analysis package IBM SPSS 24.0 (IBM, Armonk, NY, USA). The latter were carried out using the content analysis technique, which allowed us to detect the different topics that came up during the interviews for further categorization.

### 2.2. Results

The mean scores and standard deviations for each employability variable were evaluated. We obtained high scores on employment protective behavior, self-learning, and employability perception, but not on job-seeking behavior and self-control. In addition, the employment risk level was high (see Table 1).

Afterwards, a content analysis was carried out to analyze the needs and intervention strategies extracted from the interviews. Table 2 shows the categories and their frequencies.

Table 2 shows the main categories detected during the interviews. They consisted of job-seeking expectations and job-seeking behavior deficits, followed by poor self-control and job-interview coping. Finally, we found low levels of education and economic stability.

Accordingly, based on the data obtained in the previous analyses, the main elements to consider in our intervention were, on the one hand, employment risk perception, job-seeking behavior, and self-control and, on the other hand, management of job-seeking expectations, deficits in job-seeking behavior, lack of self-control, and job-interview coping.

Based on the employability diagnosis, the following program objectives were proposed: first, to promote better self-control and job-seeking behavior; second, to reduce the employment risk level; and, third, to indirectly increase the employability perception of the users.

## 3. Study 2: Design, Implementation, and Evaluation of the Intervention

### 3.1. Method

#### 3.1.1. Participants

Of the total sample, eight refugees attended the program; the other four abandoned the intervention when it began for two possible reasons. First, the CAR is a temporary center, and so users can only stay there up to nine or 12 months. Second, other interesting activities were taking place in the center related to their education, which prevented attendance. 

The intervention group was composed of eight users; the average age was 24.88 years (SD = 4.73), ranging between 20 and 33 years; 37.5% were women (*n* = 3, M_age_ = 26.75, SD = 4.57). Regarding the educational level, 75% had no education, and the remaining 25% had university studies. Moreover, 50% of these women were single, and the others were married. The other half of the total sample were men (*n* = 4, M_age_ = 23, SD = 4.69). In this case, regarding the educational level, 75% had secondary studies, and the remaining 25% had university studies. In addition, 75% were married, and the other 25% were single.

#### 3.1.2. Instruments

The same instruments used in Study 1 were used for the post-evaluation.

#### 3.1.3. Data Analysis

The statistical analysis package SPSS 24.0 was used. A quasi-experimental design was employed through a unique case design. Therefore, the design only included the intervention group. This group was evaluated twice, pretest (Study 1) and post-test (Study 2). To evaluate the P.E.R.’s effectiveness in developing skills and competences associated with employability, nonparametric tests were applied—due to the normality assumption breach—using a Mann–Whitney U test. Moreover, an effect size analysis was performed, using Cohen’s d, to discover the program’s impact in greater detail.

#### 3.1.4. Psychosocial Intervention Program

Two fundamental criteria were followed to elaborate the employability program for refugees (PER), an empirical criterion derived from the employability descriptive analysis and an expert criterion based on the professional opinion of the technicians who worked in the CAR. The program’s main objective was to promote the employability of the center’s users.

The following content was included in the program: (a) building an adequate curriculum vitae, (b) promoting tools and strategies for active job-seeking, (c) managing personal expectations about job-seeking in a new context and country, and (d) personal and interpersonal interview skills.

The PER was based on a four-factor model proposed by De Vroome and Van Tubergen [55], although it focused more on human capital and social capital. It lasted approximately 20 h, distributed in six three-hour group sessions, two per week, plus an evaluation session. These sessions were implemented during the entire month of February 2019. Only one researcher carried out the program. At the end, the users received an attendance diploma. During the program, we worked on several topics through two techniques. In the first place, cooperative learning [64] allowed us to maximize actions related to job-seeking behavior, such as building an adequate curriculum vitae, managing realistic expectations, and knowing the main job search sources in our country. Second, role-playing [65] allowed us to practice job interview skills and competences. Table 3 shows the contents of each program session. 

The program evaluation took place in the last session and lasted approximately one hour. The CAR offered us a multipurpose classroom equipped for these types of activities. The questionnaires were manually administered in a paper and pencil format, and participants’ anonymity was respected.

### 3.2. Results

The mean scores and standard deviations corresponding to the pretest and post-test assessments of each evaluated variable are presented in Table 4. The evaluated variables did not follow a normal distribution, and so nonparametric tests were applied (Table 5). The Mann–Whitney U test results showed that there were statistically significant differences associated with the job-seeking behavior (Z = −2.92, *p* = 0.003, confidence interval = 0.001, 0.003) and with emotional self-control (Z = −2.22, *p* = 0.026, confidence interval = 0.023, 0.030). In other words, the P.E.R.’s application produced an improvement in self-control and job-seeking behavior. However, no statistically significant results were obtained for the other variables.

**Figure 1:**
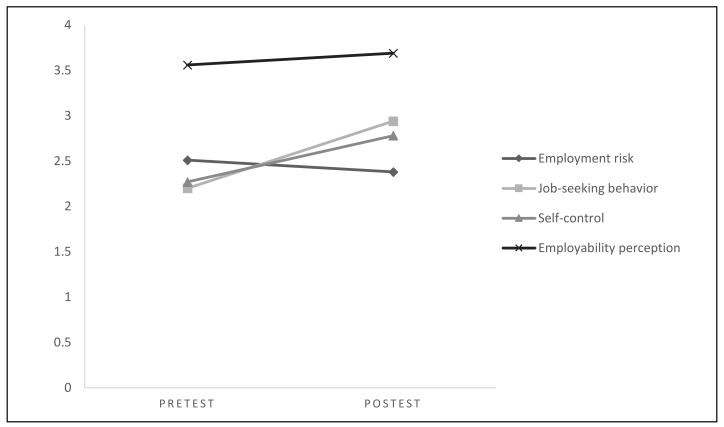
Pretest and post-test direct scores.

The differences between the pretest and post-test scores are presented in Figure 1. All scores were higher at post-test, but the employment risk decreased, as intended.

Subsequently, we analyzed the data using Cohen’s d statistic, in order to quantify the relevance of the obtained effect. High magnitudes were obtained for the job-seeking behavior and self-control variables, d = 1.85 and d = 1.14, respectively. Conversely, we obtained smaller magnitudes for the results of the other variables.

## 4. Discussion

The main objective of this research was to analyze refugees’ employability and then design, implement, and evaluate an intervention program to improve their employability skills. 

The active inclusion of refugees in the labor market is the way to modify their socioeconomic status [3] and, hence, their well-being. In this regard, some official organisms emphasize employability as a fundamental strategy to cope with poverty. However, although there are 70 million refugees in the world [25], few studies have shown interest in them. It is likely that refugees’ social barriers, such as legal regulations or missing employability skills [33], have been the “missing link” [34], and refugees have been excluded from participating in labor market integration [26]. This corresponds to an important increase in the poverty risk rate [41], which has been called the refugee crisis [42]. Therefore, our first aim was to discover refugees’ employability by evaluating them in a Refugee Reception Center in Spain. We diagnosed the refugees’ employability, and we also interviewed the institution’s social agents. 

The group of refugees was heterogeneous with diverse backgrounds. Refugees do not speak fluent Spanish, they are not familiar with Spanish culture, and they do not have an official academic degree. They obtained high scores on employment protective behavior, self-learning, and employability perception, but not on job-seeking behavior and self-control. Thus, the refugees from the CAR are persistent people, and they have the necessary personal and organizational skills for labor inclusion. However, they need to improve their active job-seeking behavior and emotional control.

The content analysis of the interviews highlighted important deficits in job-seeking expectations, job-seeking behavior, self-control, and job-interview coping. Finally, we also found low education and economic stability levels.

As the literature points out, employability is an indicator of well-being [43] because, from a holistic framework [18], it integrates characteristics such as initiative, learning to learn, autonomy, and personal care. Indeed, some authors (e.g., [55,56,57]) state that knowledge of the host country’s language, education stimulation, development opportunities, and contacts with associations or institutions and the native population, among other things, play a fundamental role in economic and labor integration. Therefore, our second aim was to develop and implement an effective and efficacious psychosocial intervention program to improve refugees’ employability skills in Spanish.

Thus, based on the employability diagnosis, we designed a program to promote better self-control and job-seeking behavior. Moreover, we wanted to reduce the employment risk level and, indirectly, increase the employability perception of the users. This program was based on the four-factor model proposed by De Vroome and Van Tubergen [55]. The results showed the program’s effectiveness in improving the self-control and job-seeking capacity. However, no differences were obtained in the employment risk level or the employability perception. 

Due to the lack of these kinds of interventions in the literature, we cannot make any comparisons, but we can highlight the importance of these measures for enhancing the collective’s social integration in our society [50]. Furthermore, it is essential to consider the four elements proposed by De Vroome and Van Tubergen [55], which include human and social capital, health, and host country policies. 

### 4.1. Limitations and Future Research

The program improved the refugees’ employability, which was our main objective. However, several limitations should be acknowledged. First, we used two employability questionnaires to measure the construct. Nevertheless, the Spanish level of some users did not allow them to answer the questions properly. Given this situation, the questionnaire was administered as a structured interview. Therefore, future intervention programs should measure employability with qualitative, observable indicators and should consider creating a standardized questionnaire that measures refugees’ employability.

Second, another limitation was the sample composition. The intervention program was designed for refugees who were in a specific CAR, and there were few registered users. Thus, the sample size was reduced, which may have affected the results obtained. In this sense, Bagiella and Chang [66] state that intervention studies with small sample sizes may not have statistically significant effects even if they exist and could refuse interventions that actually generate a real effect on people. However, many intervention programs have this limitation because they are intended for specific groups and, in this sense, resemble case studies. The aim of this study is not to generalize the results, mainly because the sample only pertains to a working-age population. Nevertheless, future studies should increase the number and ages of subjects so as to reach a statistical power sufficient for detecting the magnitude of the effect of the intervention program. 

Third, the SPSS statistical package and the techniques we used required the use of large samples. Thus, future studies should consider the use of statistical packages that are not susceptible to the sample size or the use of qualitative techniques. 

Fourth, due to the characteristics of the sample and the temporality problem, only two measures were taken (pretest and post-test). These measures are considered sufficient to evaluate intervention programs. Nevertheless, future interventions should be designed with a greater time extension that allows three or more evaluation measures.

Fifth, the intervention program focused on human and social capital. It did not consider other components proposed by De Vroome and Van Tubergen [55], such as health problems. However, due to the short stay, we preferred to focus on only a few aspects in order to produce better effects. Future research should consider aspects such as war experiences, cultural aspects, etc. It is in line with the notion of social investment to improve the employability of disadvantaged groups [67], as well as the job security [68] and the health and well-being of refugees [69]. 

Despite these limitations, the results showed the efficacy of the intervention program in improving the refugees’ employability capacity. Given that employability is fundamental to social integration [70], implementing these types of measures could be essential in achieving this objective. By carrying out these initiatives in a coordinated and integrated way with the different associations or centers belonging to the Employment and Social Security Ministry, we could improve refugees’ risk of social exclusion and promote their psychosocial integration.

### 4.2. Implications

Thus, this program represents an advance in the refugees’ social and labor inclusion process, and it has theoretical and practical implications. As regards theoretical implications, it should be noted that we did not find data on the individual employability variables of refugees in Spain. The program also has practical implications because it was an attempt to promote refugees’ labor integration in Spain. So far, only a few isolated initiatives have pursued refugees’ integration in the Spanish context (such as Altius Foundation with the Erasmus + Restore Respect project) [71].

We would also like to highlight the practical policy implications derived from our results because the political and social debate about population displacement is not new and implies major challenges for all states [25]. Indeed, in 2018 the Global Refugee Pact was signed by the ONU General Assembly. This is a nonbinding pact, but it implies a commitment to implement migration policies of social and labor inclusion for refugees. However, the access of refugees to the most basic social services (e.g., housing, employment, education, health...) is a difficult matter [72]. The labor inclusion of refugees is a long-term goal that is difficult to implement. 

In Spain, the process of integration has been slow. The right to work is developed in a context of crisis and precarious markets. This implies the need for new models and best practices. In this sense, there is a demand in Spain for policies that refer to alternative methods that evaluate and promote not only the transversal but also the technical and linguistic skills of refugees. In the current business context, human resource policies are based on skills management, and therefore their development must be part of the public policies that contribute to access to employment. This development can be promoted at an individual or group level, as in the intervention program developed in this study, and can focus on the development of transversal and/or technical competencies that depend on the characteristics of the group in question.

## 5. Conclusions

Refugees have a high unemployment rate and difficult access to employment [73]. However, the intervention program carried out following De Vroome and Van Tubergen’s theory and the bioecological perspective of employability [18] emerges as an innovative tool in the job-training field that could provide vulnerable groups with strategies to access employment.

## Figures and Tables

**Table 1:** Descriptive statistics.

Dimensions	M	SD
Employment protective behavior	4.12	0.42
Employment risk	2.51	0.42
Job-seeking behavior	2.2	0.52
Self-control	2.27	0.39
Self-learning	4.36	0.78
Employability perception	3.56	0.51

Note: M = Mean, SD = Standard Deviation.

**Table 2:** Frequencies.

Dimensions	f_i_	n_i_
Job-seeking expectations	3	0.25
Job-seeking behavior deficits	3	0.25
Specific education	1	0.08
Job-interviews coping	2	0.17
Economic stability	1	0.08
Poor self-control	2	0.17

Note: n_i_ = Relative frequency, f_i_ = Absolute frequency.

**Table 3:** Content of each session and number of participants.

Activities	Content
Curriculum Vitae (CV)	Motivation. Positive interaction. CV’s preparation. Assertiveness.
Employability expectations	Knowledge about the Spanish labor market. Reflection. Realistic expectations.
Job-seeking sources	Active seek. Knowledge about job-seeking sources. Stress/frustration management.
Interview questions	Active seek. Knowledge about job-seeking sources. Stress/frustration management.
Interview abilities	Interview abilities. Verbal and nonverbal behavior in the interview.
Putting into practice	Interview abilities. How to answer questions. Verbal and nonverbal behavior in the interview.

**Table 4:** Descriptive statistics.

Dimensions	Pretest			Post-Test		
	M	SD	*n*	M	SD	*n*
Employment risk	2.51	0.42	11	2.38	0.53	8
Job-seeking behavior	2.2	0.52	11	2.94	0.23	8
Self-control	2.27	0.39	11	2.78	0.49	8
Employability perception	3.56	0.51	12	3.69	0.77	8

Note: M = Mean, SD = Standard Deviation, *n* = total number of individuals or cases.

**Table 5:** Normal distribution tests.

Dimensions	Skewness		Kurtosis		K-S Test	
	Sk	SE	K	SE	Z	*p*-Value
Employability	−0.9	0.52	0.77	1.01	0.14	0.200
Employability perception	1.09	0.51	0.24	0.99	0.27	0.000

Note: Sk = Skewness, SE = Standard Error, K = Kurtosis, Z = Z-test
